# Supplementary material for: Twin boundary migration in an individual platinum nanocrystal during catalytic CO oxidation
Source: Nat Commun. 2021 Sep 10;12:5385. doi: 10.1038/s41467-021-25625-0 (PMC8433154; doi:10.1038/s41467-021-25625-0)
Supplement: Supplementary file 1 — Supplementary Information [file 41467_2021_25625_MOESM1_ESM.pdf]

Supplementary Material: Twin boundary migration in an individual platinum nanocrystal during catalytic CO oxidation

Jérôme Carnis[1,2,#], Aseem Rajan Kshirsagar[3], Longfei Wu[1,2], Maxime Dupraz[1,2], Stéphane Labat[1], Michaël Texier[1], Luc Favre[1], Lu Gao[4], Freddy E. Oropeza[4], Nimrod Gazit[5], Ehud Almog[5], Andrea Campos[6], Jean-Sébastien Micha[7], Emiel J. M. Hensen[4], Steven J. Leake[2], Tobias U. Schülli[2], Eugen Rabkin[5], Olivier Thomas[1], Roberta Poloni[3], Jan P. Hofmann[4,$] and Marie-Ingrid Richard[1,2,&*]

[1] *Aix Marseille Université, CNRS, Université de Toulon, IM2NP UMR 7334, 13397, Marseille, France.*
[2]*ID01/ESRF, The European Synchrotron, 71 Avenue des Martyrs, 38000 Grenoble, France.*
[3]*Grenoble-INP, SIMaP, University of Grenoble-Alpes, CNRS, F-38042 Grenoble, France.*
[4]*Laboratory for Inorganic Materials and Catalysis, Department of Chemical Engineering and Chemistry, Eindhoven University of Technology, P. O. Box 513, 5600 MB Eindhoven, The Netherlands.*
[5]*Department of Materials Science and Engineering, Technion-Israel Institute of Technology, 3200003, Haifa, Israel.*
[6]*Aix Marseille Univ, CNRS, Centrale Marseille, FSCM (FR1739), CP2M, 13397 Marseille, France*
[7]*CRG-IF BM32 beamline at the European Synchrotron (ESRF), CS40220, 38043 Grenoble Cedex 9, France.*
[#]*Now, at Deutsches Elektronen-Synchrotron (DESY), Notkestraße 85, 22607 Hamburg*
[&]*Now, at Univ. Grenoble Alpes, CEA Grenoble, IRIG, MEM, NRS, 17 rue des Martyrs 38000 Grenoble, France*
[$]*Now, at Surface Science Laboratory, Department of Materials and Earth Sciences, Technical University of Darmstadt, Otto-Berndt-Strasse 3, 64287 Darmstadt, Germany*

Condition	Temperature	Gas flows
1	450°C	Ar
2	450°C	Ar and CO (2% in Ar)
3	450°C	Ar, CO (2% in Ar) and O_2 (0.4% in Ar)
4	450°C	Ar, CO (2% in Ar) and O_2 (1% in Ar)
5	450°C	Ar, CO (2% in Ar) and O_2 (4% in Ar)
6	450°C	Ar and O_2 (4% in Ar)
7	450°C	Ar
8	450°C	Ar and CO (2% in Ar)
9	500°C	Ar and CO (2% in Ar)

Supplementary Table 1: Temperature and gas conditions

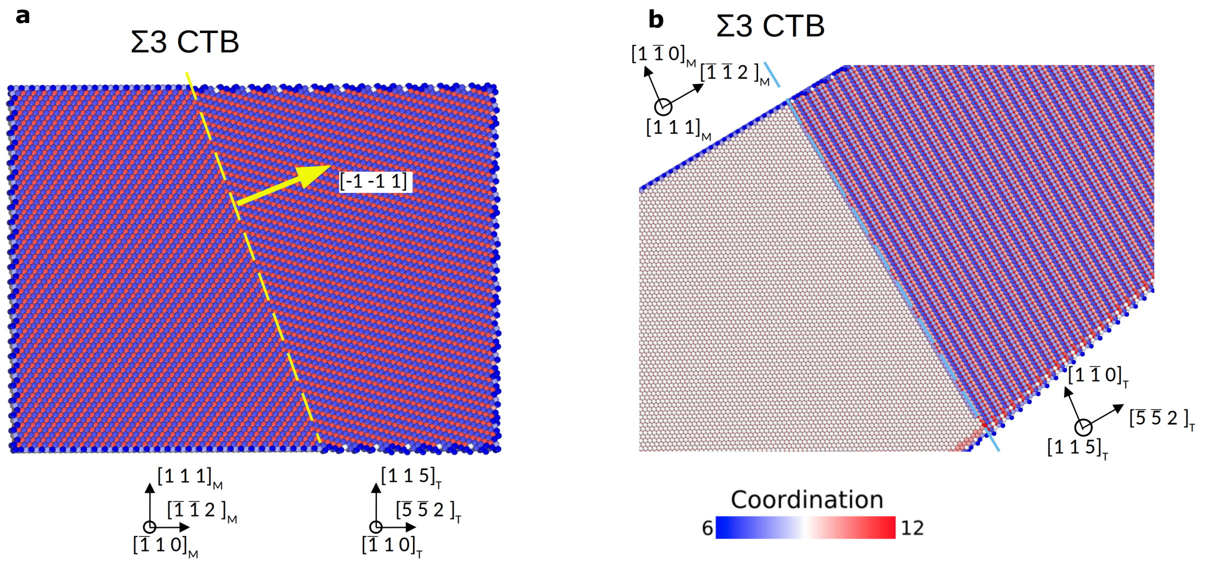

Supplementary Figure 1: Illustration of the atomistic configuration of the Σ3{111} coherent twin boundary (CTB). The atoms are colored according to their coordination numbers.

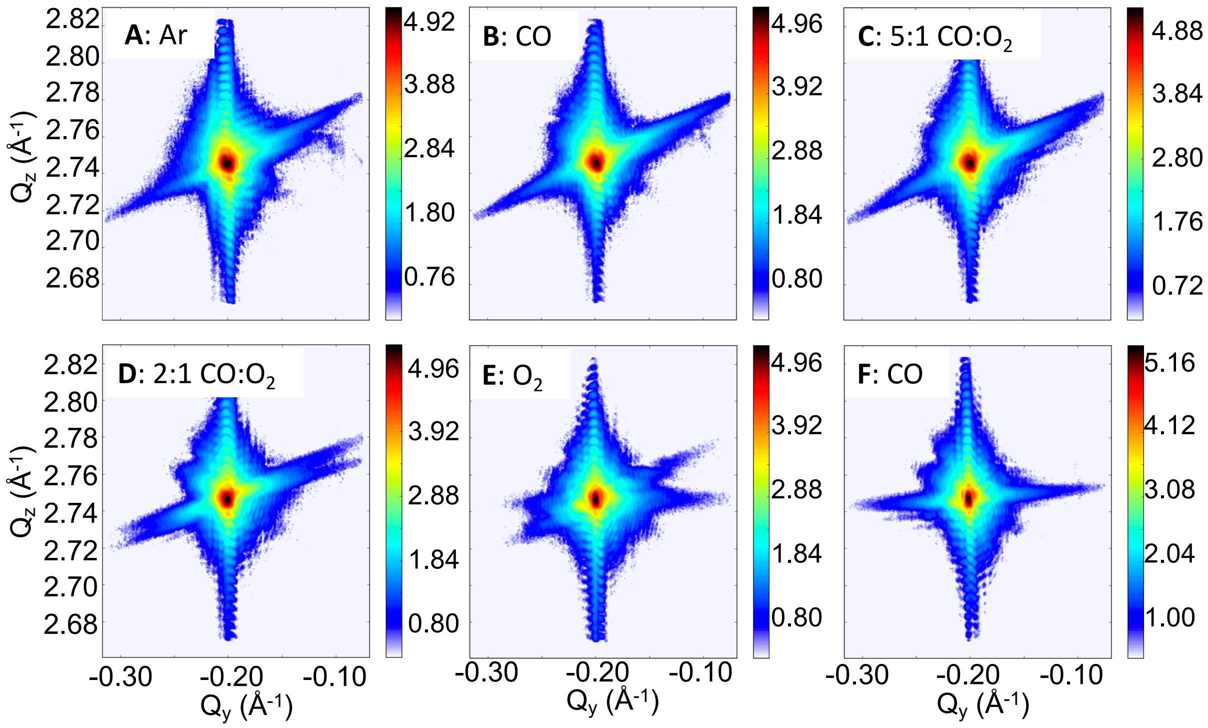

Supplementary Figure 2: 111 Pt diffraction patterns measured at 450°C, summed along the reciprocal space coordinate Q_x (Q_x being along the x-ray beam). The gas composition is indicated in the top left corner.

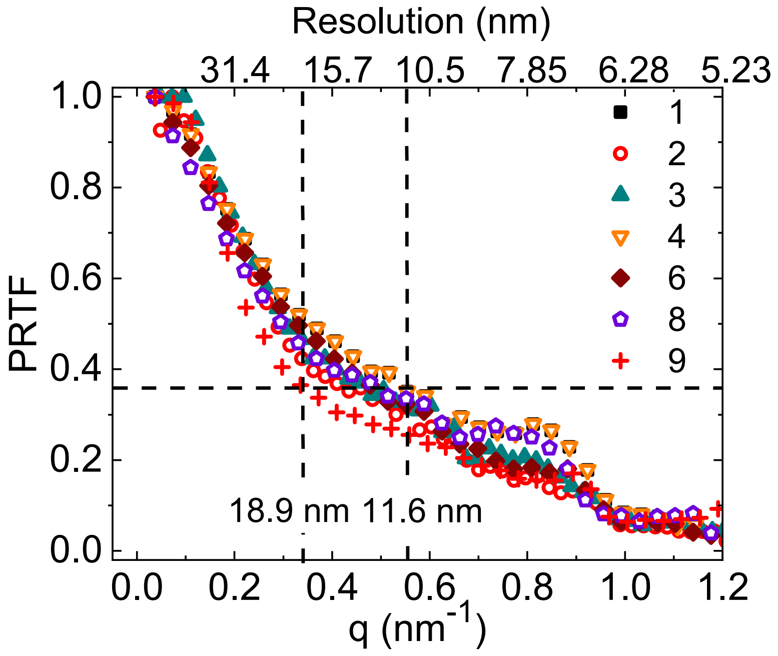

Supplementary Figure 3: Spatial resolutions obtained by the phase retrieval transfer function (PRTF) at a cut-off of 1/*e* for different gas conditions (1, 2, 3, 4, 6, 8 and 9).

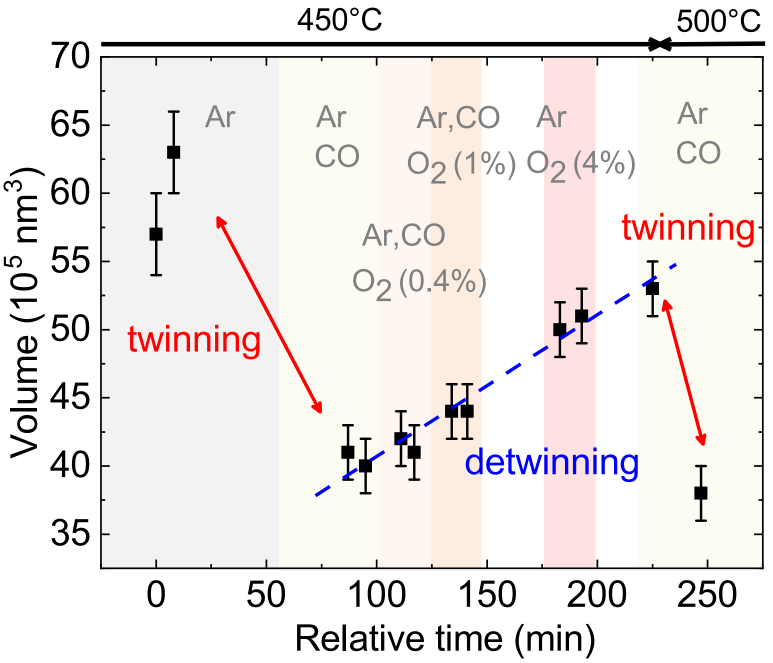

Supplementary Figure 4: Evolution of the estimated reconstructed volume (error bar corresponding to 10% of the estimated volume) as a function of the gas composition.

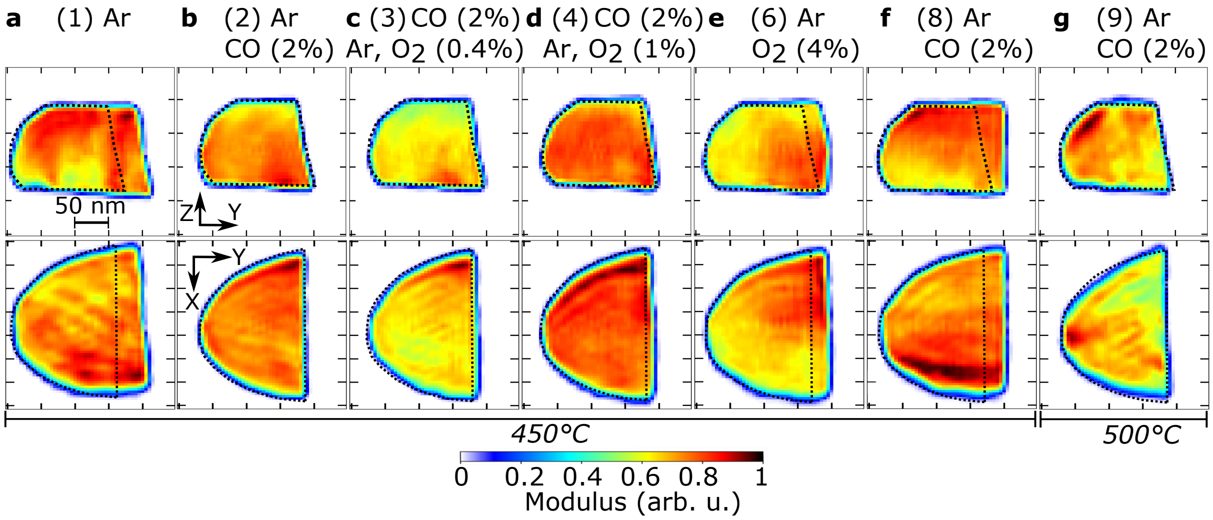

Supplementary Figure 5: 2D slices through the center of the reconstructed modulus of the crystal. The dotted black contours are guide for the eyes and delimit the shape of the twinned crystal measured at condition (2): Ar and CO (2%) at 450°C. Tick spacing corresponds to 50 nm.

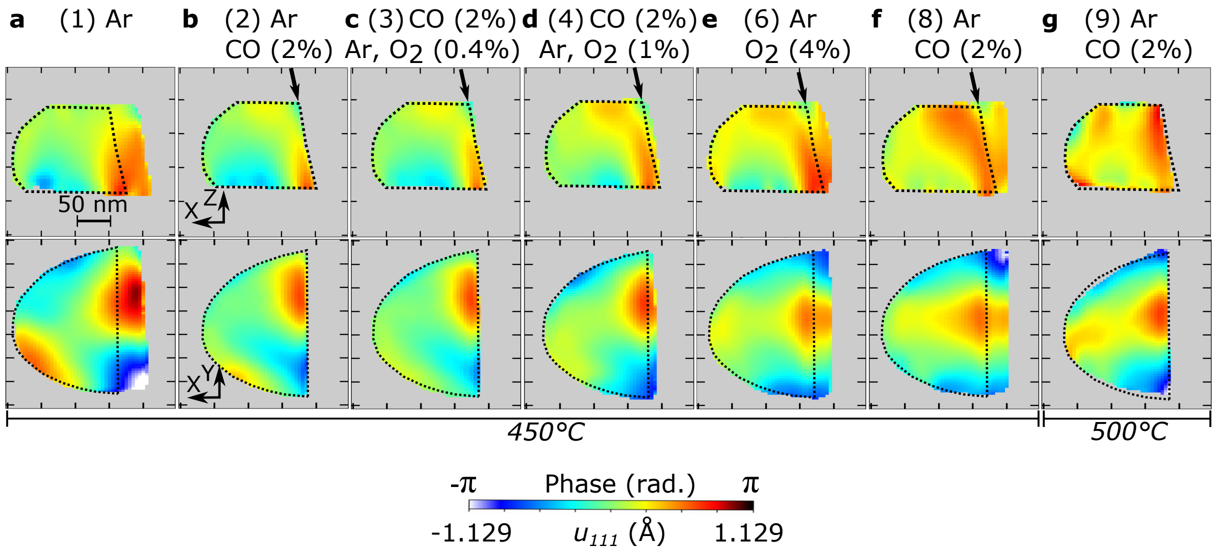

Supplementary Figure 6: 2D slices through the center of the reconstructed phase or displacement field along the [111] direction of the crystal. The dotted black contours are guide for the eyes and delimit the shape of the twinned crystal measured at condition (2): Ar and CO (2%) at 450°C. Tick spacing corresponds to 50 nm.

XCF	$\gamma_{hkl}^{vac}(J/m^2)$ 111	511	100
revPBE	1.343	1.618	1.657
revPBE-D3	2.469	2.769	
rev-vdW-DF2	1.783	2.223	2.160
LDA (earlier work[a])	1.992 (1.98)	2.374	(2.35)
PBE (earlier work[a])	1.509 (1.56)	1.806	1.836 (1.88)
PBE-D3[b]	2.240	2.529	2.513
PBE-D3	2.185	2.515	
PBEsol	1.852 (1.85)	2.222 (2.21)	
SCAN+rvv10 (earlier work[a])	1.89		2.25
Experiment[c]	2.49		

Supplementary Table 2. Surface energies of different Pt surfaces calculated with a range of XCFs. revPBE has been chosen due to its accuracy in predicting adsorption energies of molecules on metal surfaces [1]. 'D3' refers to the Grimme's DFT-D3 van der Waal's correction without damping as implemented in QE. [a] From reference [2]. [b] With PBE geometry. [c] From reference [3, 4].

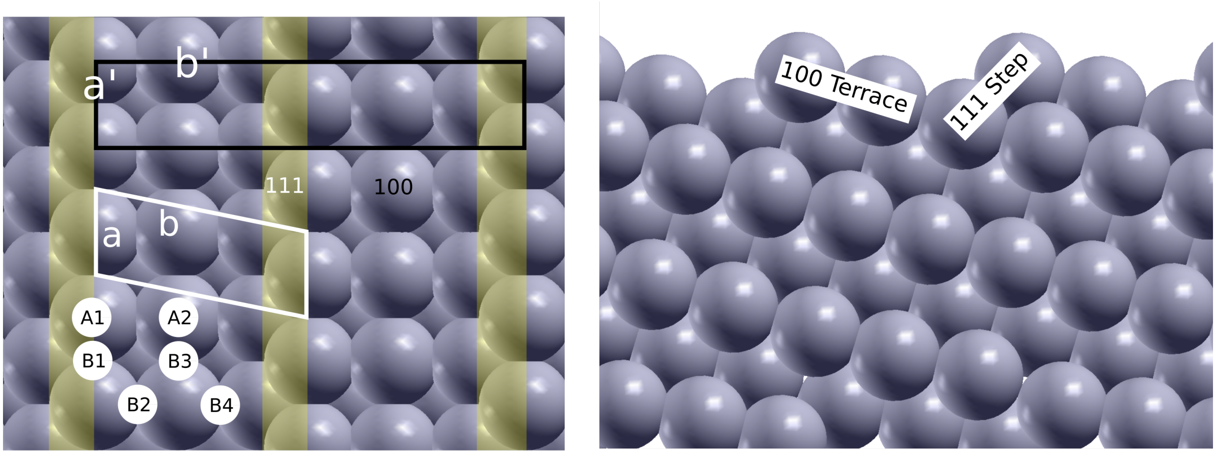

(a) (b)

Supplementary Figure 7: (a) Top view of Pt {511} surface. White and black solid lines show the primitive and unit cell, respectively. The ***a'***, ***b'*** in-plane unit cell vectors of the unit cell used here (black lines) are defined as ***a'***=***a*** and ***b'*** = 2***b***-***a***, where ***a*** and ***b*** refer to the primitive cell. The yellow region shows the {111} step and the rest of the area corresponds to {100} terrace. A1, A2, B1, B2, B3 and B4 are the distinct CO adsorption sites. The first two correspond to top sites and latter four correspond to bridge sites. Note that there are two extra adsorption sites H and B which are hollow and bridge sites on the {111} step.

XCF ↓	$\gamma^{CO}_{hkl}\,(J/m^2)$ 111		511		
Coverage →	0.108	0.144	0.108	0.120	0.144
revPBE	-0.257	-0.084	-0.694	-0.745	-0.201
revPBE-D3	-0.317	-0.436	-0.636	-0.729	-0.474
rev-vdW-DF2	-0.835	-0.994	-1.013	-1.158	-0.843
LDA	-1.827	-2.117	-2.04	-2.319	-2.209
PBE	-0.819	-0.700	-1.105	-1.147	-0.791
PBE-D3	-0.630	-0.989	-0.940	-1.035	-0.747
PBEsol	-1.259	-1.272	-1.468	-1.619	-1.346

Supplementary Table 3. CO/Pt interfacial energy computed using different choices of the XCF at varying CO coverages (CO/Å^2). We note that regardless of the functional choice, the CO/Pt interfacial energy is always lower for {511} than {111} at high coverages.

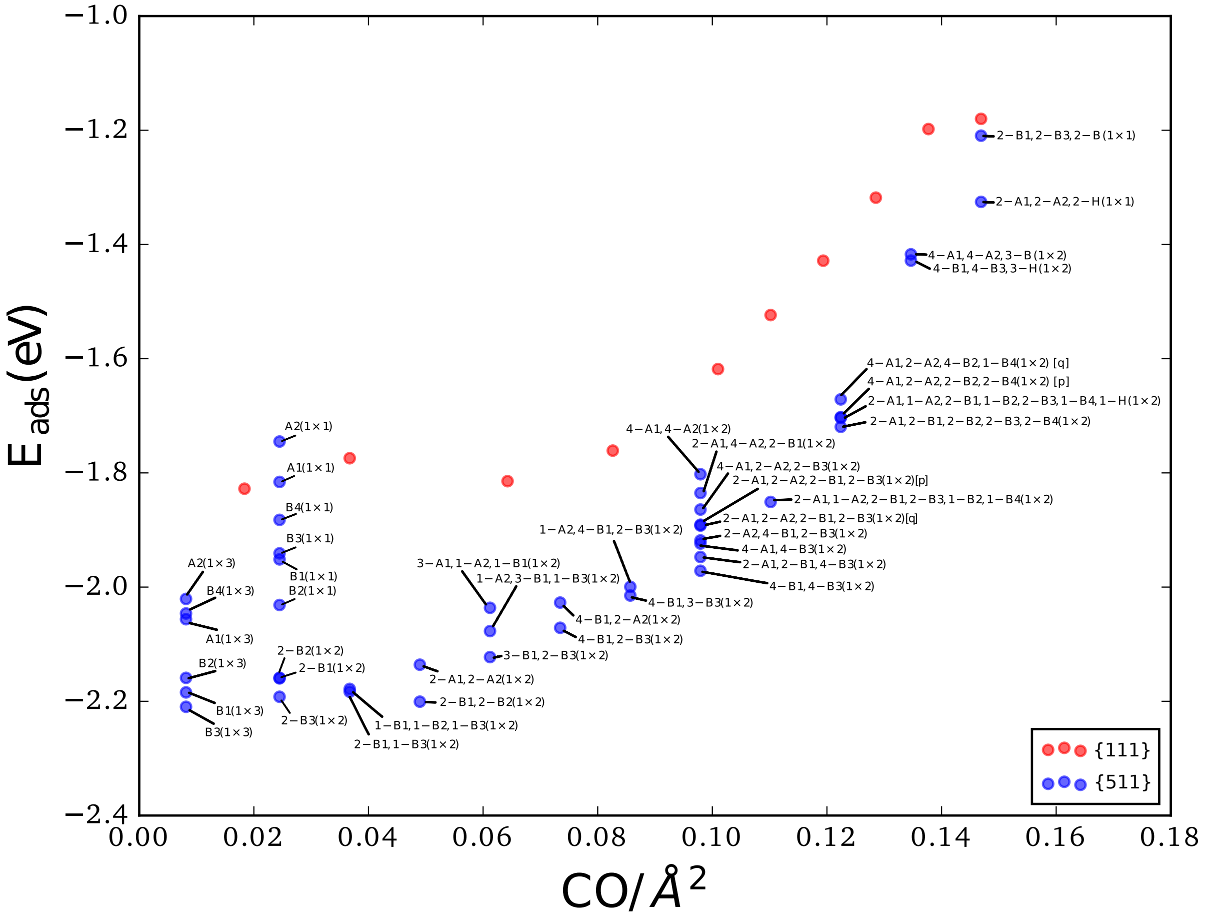

Supplementary Figure 8: CO adsorption energy on {111} and {115} surfaces at different CO coverages computed using rev-vdW-DF2. The configurations of adsorbed CO on {111} are taken from Ref. [5]. The nomenclature used to describe CO on {511} is described in Figure S6.

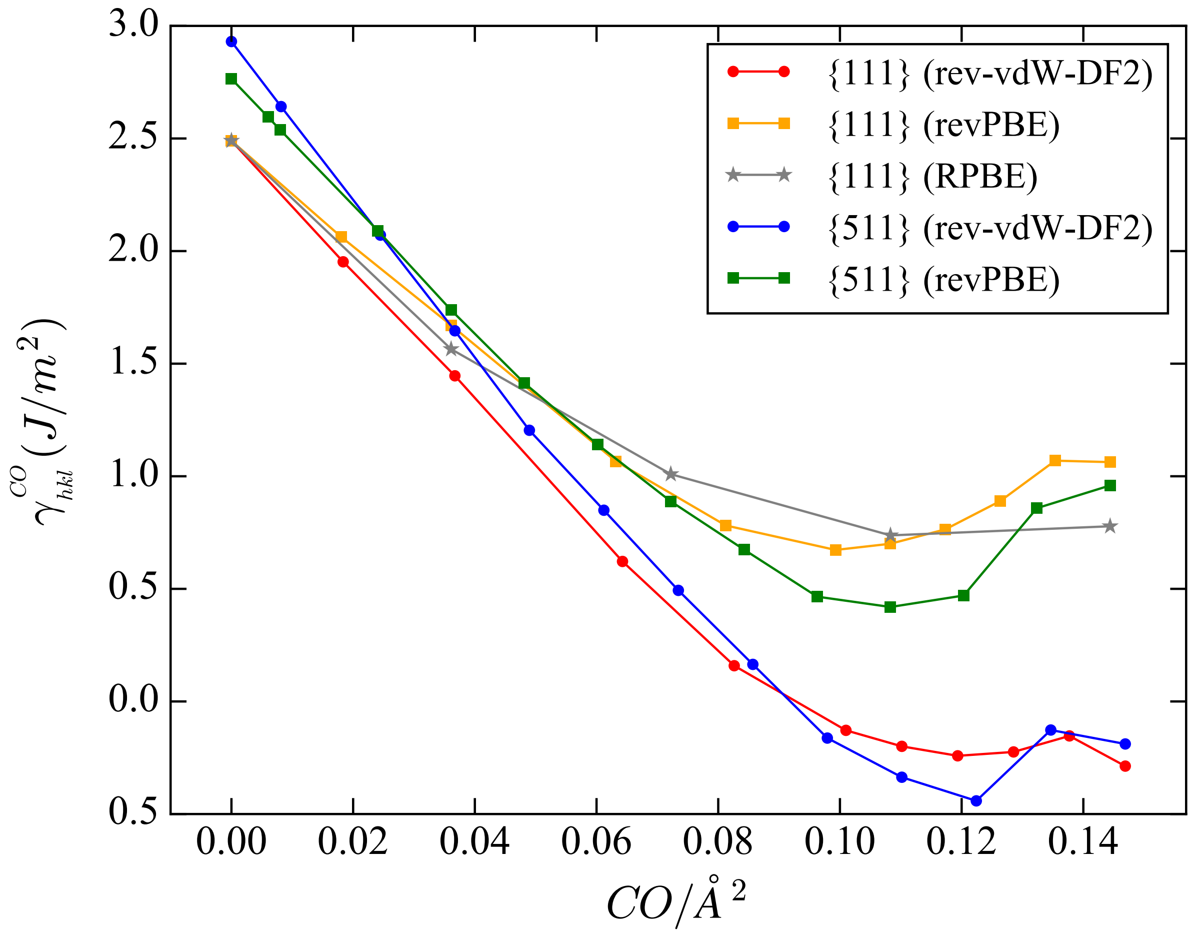

Supplementary Figure 9: Interfacial energy as a function of CO coverage for different XCFs. The γ_{hkl}^{CO} curves plotted here are shifted by a constant such that the experimental surface energy of {111} is recovered for γ_{111}^{vac}. Specifically, a functional-dependent rigid shift is applied to the values reported in Table S3. Once the shift is determined for γ_{111}^{vac}, the same value is used to shift γ_{511}^{vac}. The data points for RPBE are recalculated by using results (binding energies and surface energies) from an earlier work [6].

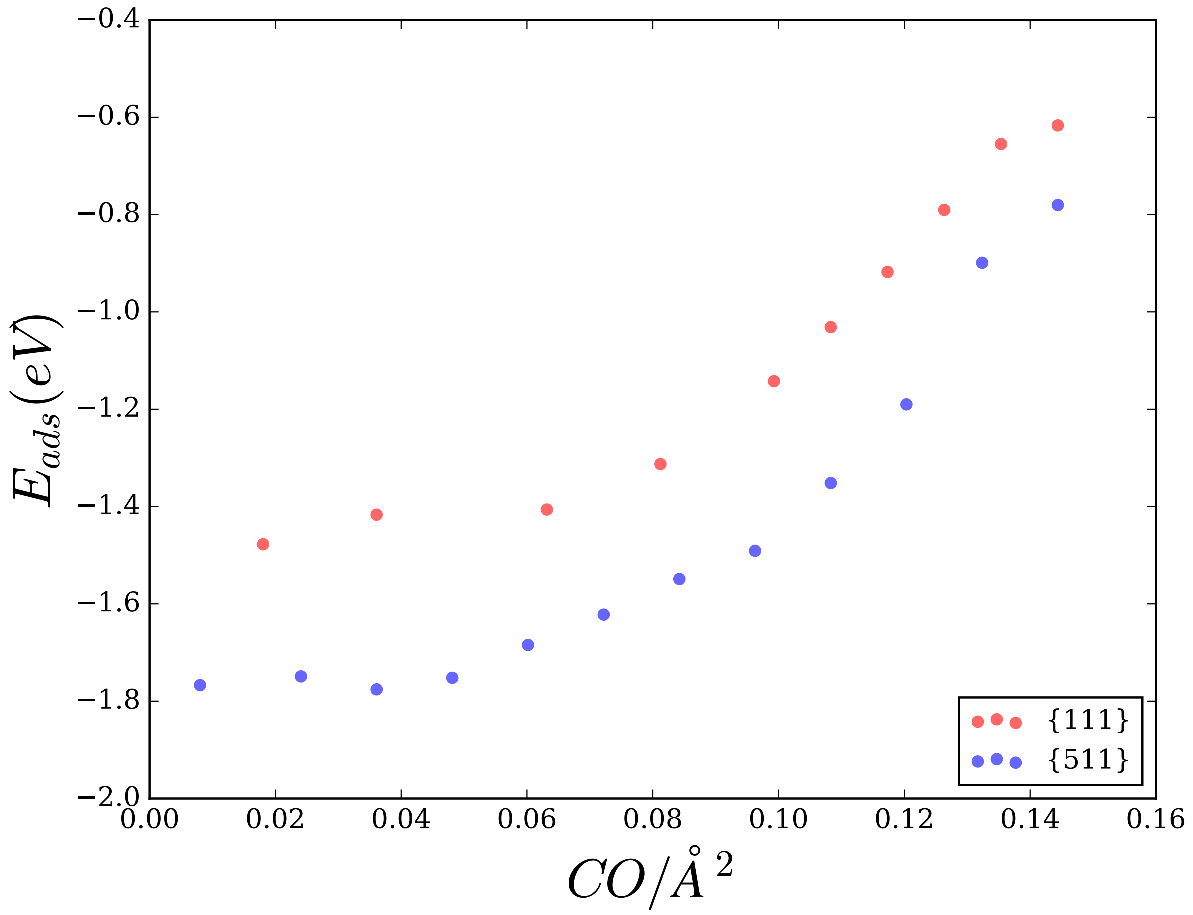

Supplementary Figure 10: CO adsorption energy on {111} and {115} surfaces at different CO coverages computed using revPBE. For {111}, the CO configurations are taken from Ref. [5]. For {511} the lowest energy ones are taken from calculations shown in Figure S7.

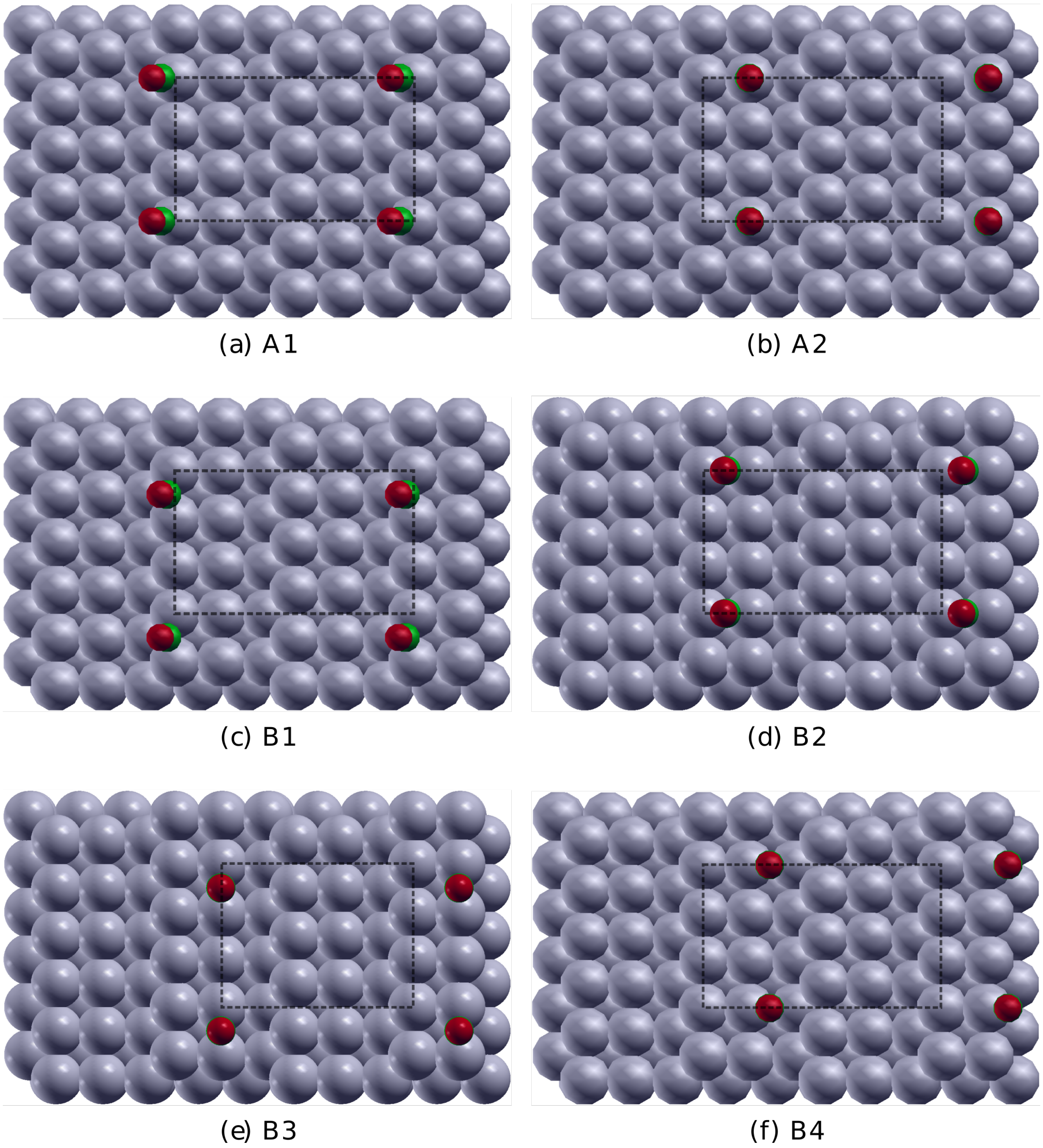

Supplementary Figure 11: Illustration of the CO/Pt{511} configurations computed in this work corresponding to a coverage of 0.008161 CO/Å^2 (*i.e.* 0.056 ML) using a 1×3 supercell.

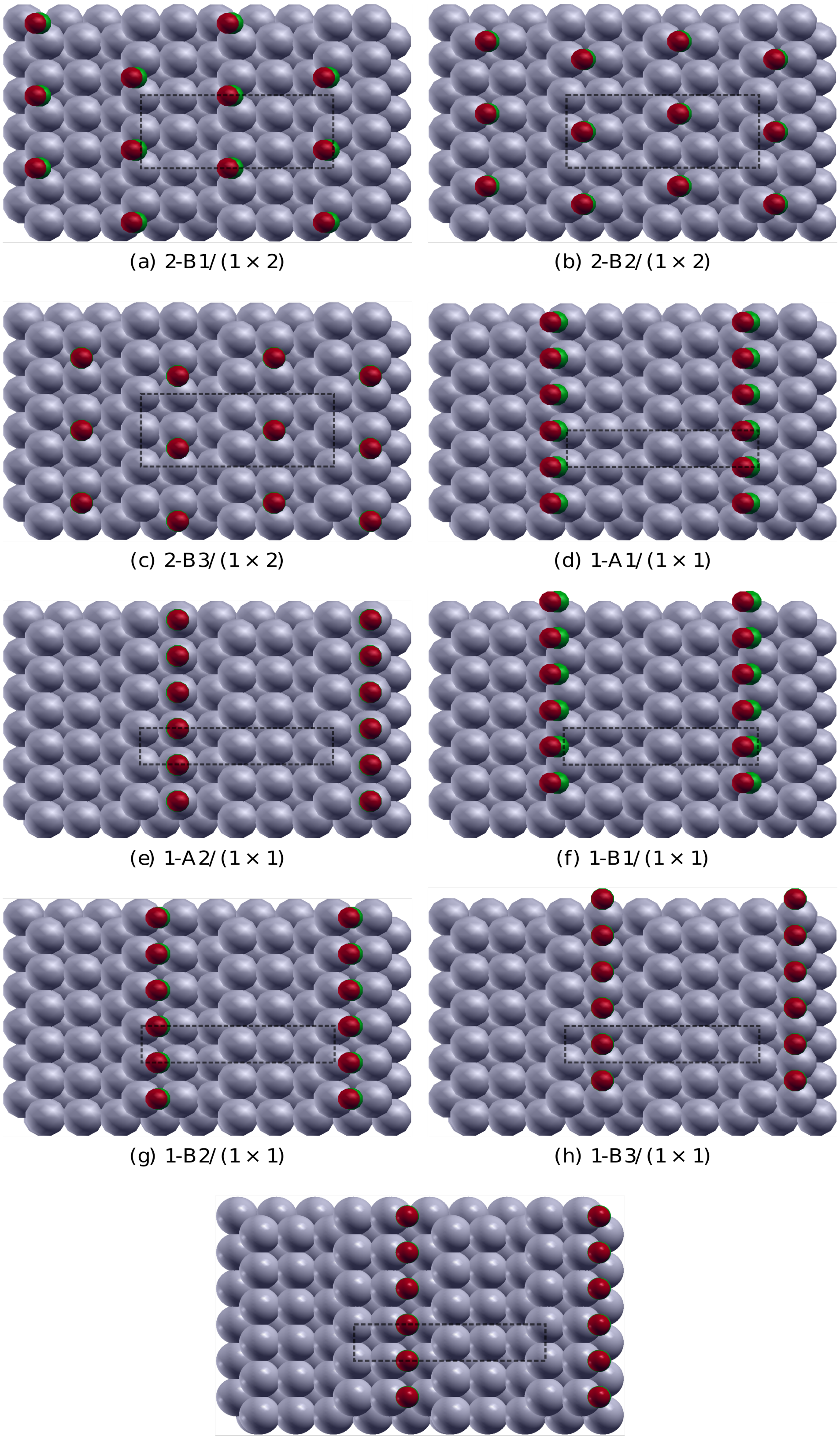

(a) 2-B1/(1 × 2)
(b) 2-B2/(1 × 2)
(c) 2-B3/(1 × 2)
(d) 1-A1/(1 × 1)
(e) 1-A2/(1 × 1)
(f) 1-B1/(1 × 1)
(g) 1-B2/(1 × 1)
(h) 1-B3/(1 × 1)
(i) 1-B4/(1 × 1)

Supplementary Figure 12: Illustration of the CO/Pt{511} configurations computed in this work corresponding to a coverage of 0.024482 CO/$Å^2$ (*i.e.* 0.166 ML) using 1×2 and 1×1 supercells.

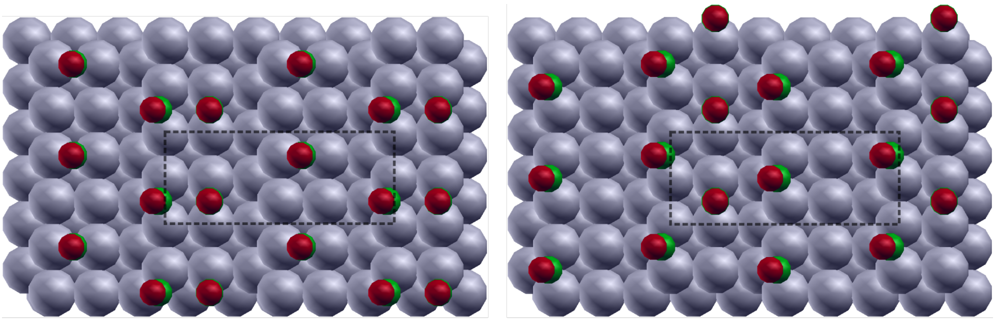

(a) 1-B1,1-B2,1-B3/ (1 × 2)   (b) 2-B1,1-B3/ (1 × 2)

Supplementary Figure 13: Illustration of the CO/Pt{511} configurations computed in this work corresponding to a coverage of 0.036724 CO/Å^2 (*i.e.* 0.250 ML) using a 1×2 supercell.

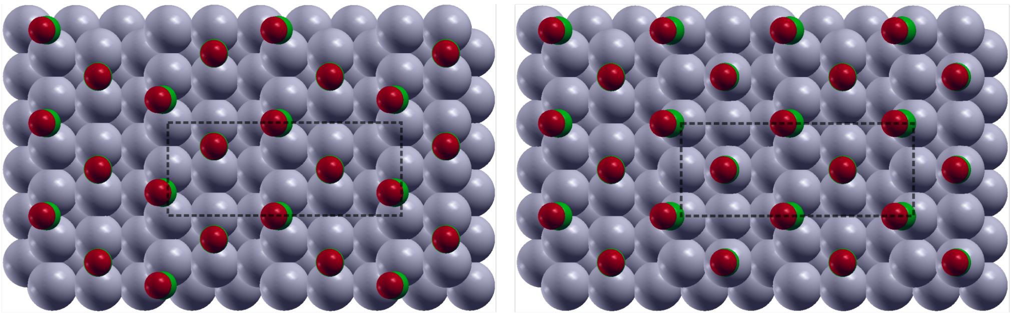

(a) 2-B1,2-B3/ (1 × 2)   (b) 1-A2,2-B1,1-B3/ (1 × 2)

Supplementary Figure 14: Illustration of the CO/Pt{511} configurations computed in this work corresponding to a coverage of 0.048965 CO/Å^2 (*i.e.* 0.333 ML) using a 1×2 supercell.

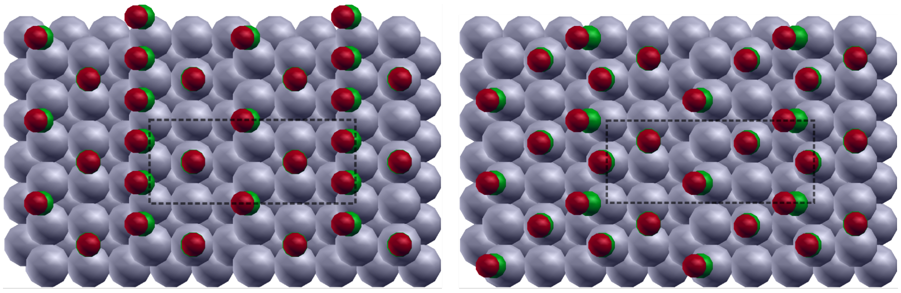

(a) 3-A1,1-A2,1-B3/ (1 × 2)   (b) 2-A1,1-A2,1-B1,1-B3/ (1 × 2)

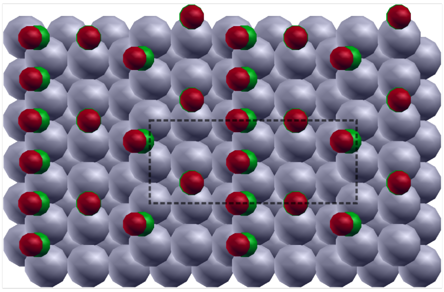

(c) 3-B1,2-B3/ (1 × 2)

Supplementary Figure 15: Illustration of the CO/Pt{511} configurations computed in this work corresponding to a coverage of 0.060168 CO/Å^2 (*i.e.* 0.416 ML) using a 1×2 supercell.

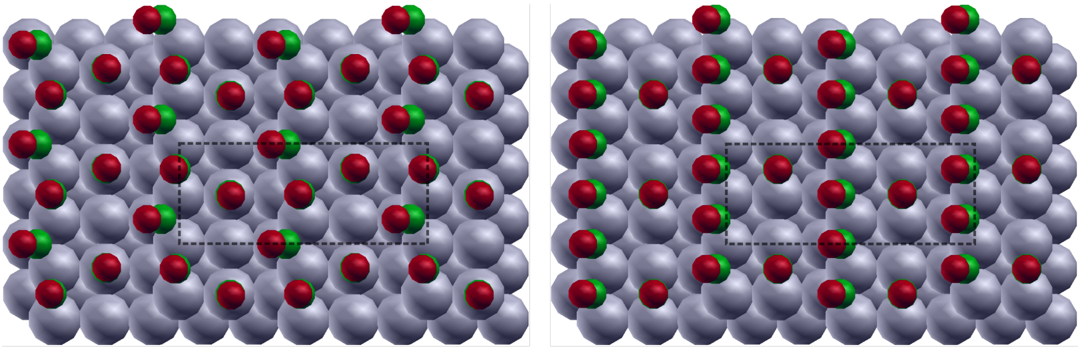

(a) 2-A2,4-B1/(1 × 2) (b) 4-B1,2-B3/(1 × 2)

Supplementary Figure 16: Illustration of the CO/Pt{511} configurations computed in this work corresponding to a coverage of 0.073447 CO/Å^2 (*i.e.* 0.500 ML) using a 1×2 supercell.

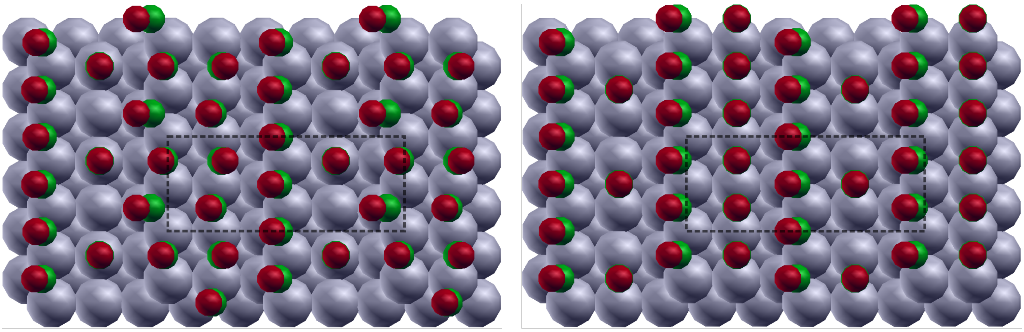

(a) 1-A2,4-B1,2-B3/(1 × 2) (b) 4-B1,3-B3/(1 × 2)

Supplementary Figure 17: Illustration of the CO/Pt{511} configurations computed in this work corresponding to a coverage of 0.085689 CO/Å^2 (*i.e.* 0.583 ML) using a 1×2 supercell.

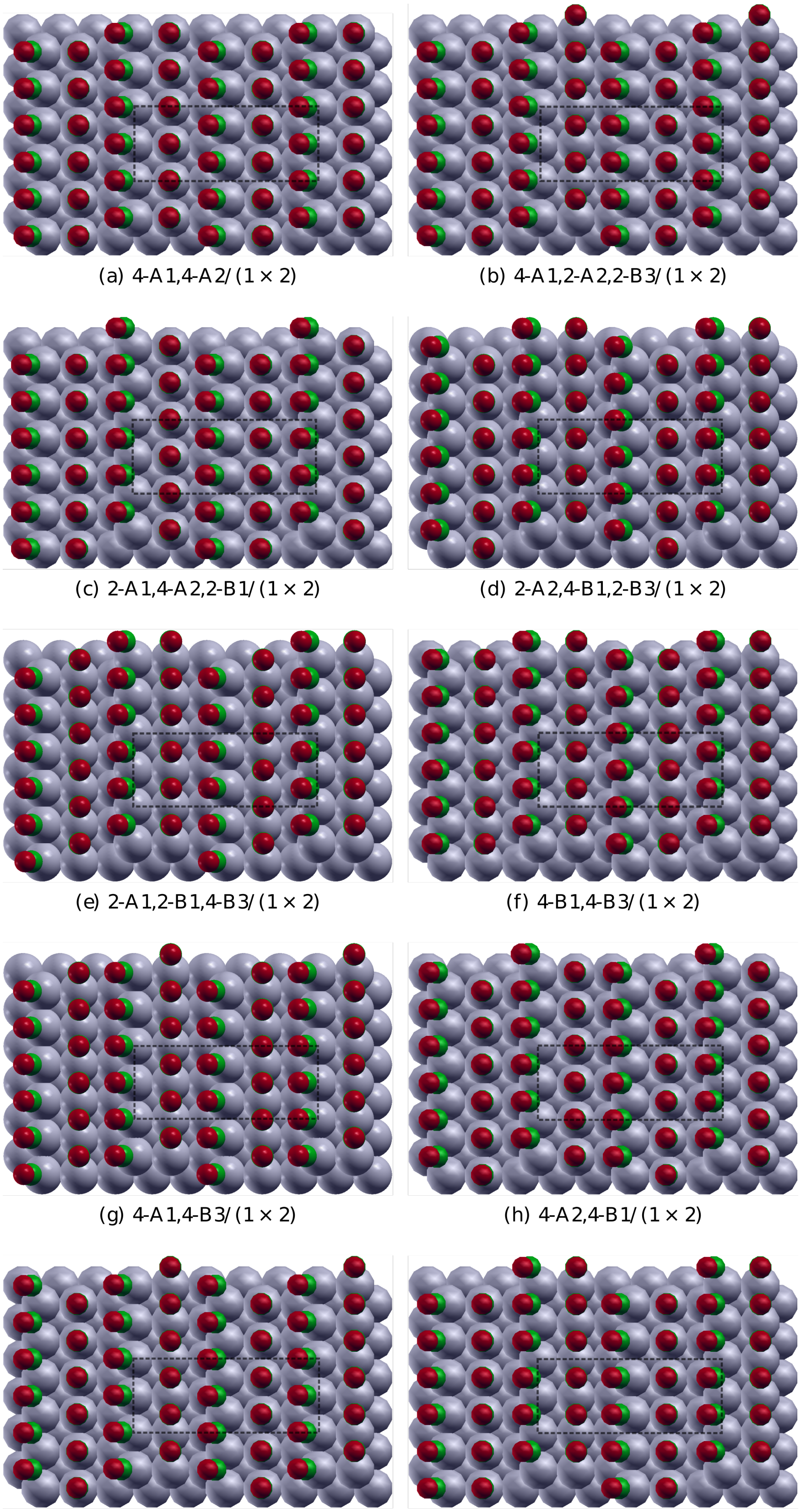

(a) 4-A1,4-A2/(1 × 2)
(b) 4-A1,2-A2,2-B3/(1 × 2)
(c) 2-A1,4-A2,2-B1/(1 × 2)
(d) 2-A2,4-B1,2-B3/(1 × 2)
(e) 2-A1,2-B1,4-B3/(1 × 2)
(f) 4-B1,4-B3/(1 × 2)
(g) 4-A1,4-B3/(1 × 2)
(h) 4-A2,4-B1/(1 × 2)
(i) 2-A1,2-A2,2-B1,2-B3 (q)/(1 × 2)
(j) 2-A1,2-A2,2-B1,2-B3 (p)/(1 × 2)

Supplementary Figure 18: Illustration of the CO/Pt{511} configurations computed in this work corresponding to a coverage of 0.097930 CO/Å^2 (*i.e.* 0.667 ML) using a 1×2 supercell.

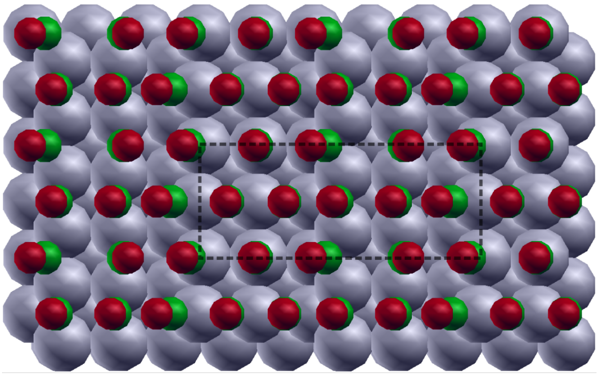

(a) 2-A1,1-A2,2-B1,2-B3,1-B2,1-B4/ (1 × 2)

Supplementary Figure 19: Illustration of the CO/Pt{511} configuration computed in this work corresponding to a coverage of 0.110171 CO/Å^2 (*i.e.* 0.75 ML) using a 1×2 supercell.

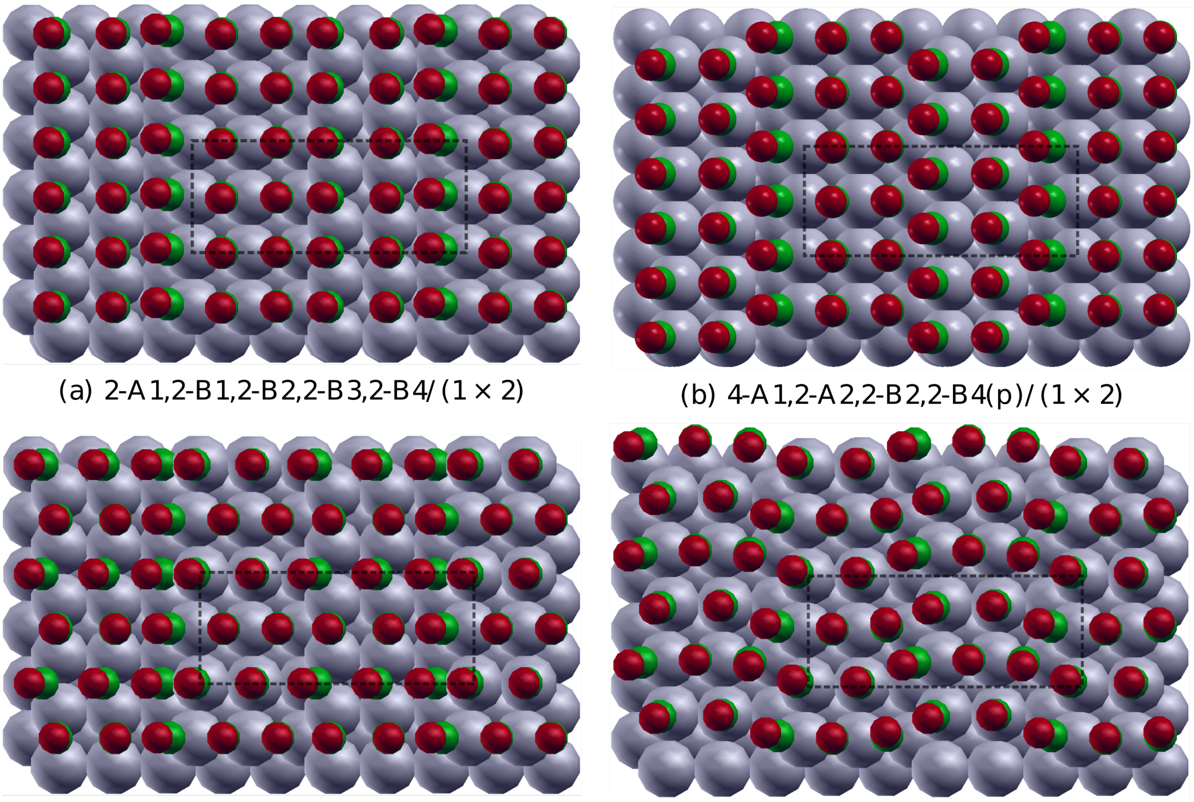

(a) 2-A1,2-B1,2-B2,2-B3,2-B4/ (1 × 2)

(b) 4-A1,2-A2,2-B2,2-B4(p)/ (1 × 2)

(c) 2-A1,1-A2,2-B1,1-B2,2-B3,1-B4,1-H/ (1 × 2)

(d) 4-A1,2-A2,2-B2,2-B4(q)/ (1 × 2)

Supplementary Figure 20: Illustration of the CO/Pt{511} configurations computed in this work corresponding to a coverage of 0.122412 CO/Å^2 (*i.e.* 0.833 ML) using a 1×2 supercell. Here, B and H are bridge and hollow sites on {111} step, respectively.

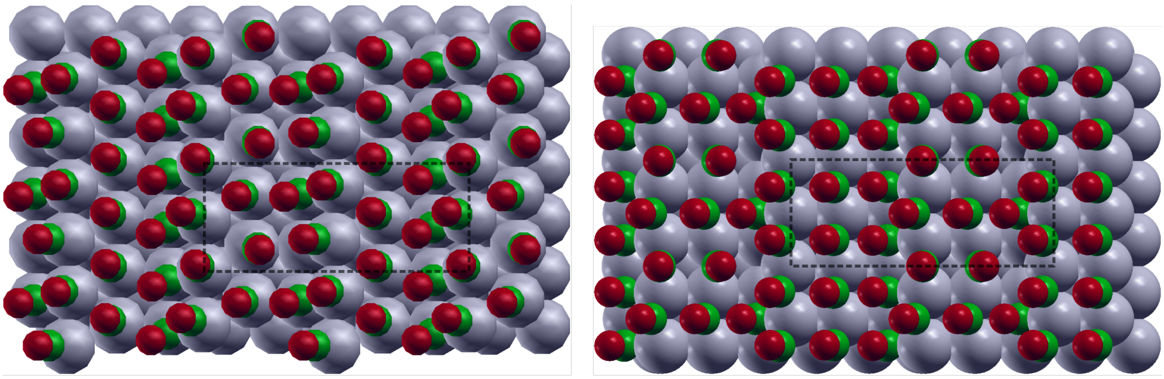

(a) 4-A1,4-A2,3-B/(1 × 2) (b) 4-B1,4-B2,3-H/(1 × 2)

Supplementary Figure 21: Illustration of the two CO/Pt{511} configurations computed in this work corresponding to a coverage of 0.134654 CO/Å^2 (*i.e.* 0.917 ML) using a 1×2 supercell. Here, B is bridge site on {111} step.

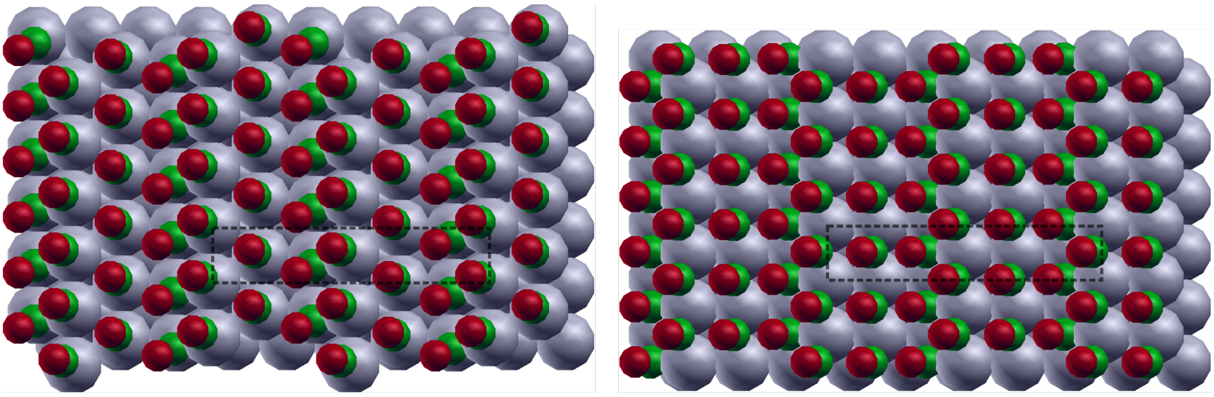

(a) 2-A1,2-A2,2-B/(1 × 1) (b) 2-B1,2-B2,2-H/(1 × 1)

Supplementary Figure 22: Illustration of the two CO/Pt{511} configurations computed in this work corresponding to a coverage of 0.146895 CO/Å^2 (*i.e.* 1 ML coverage) on the unit cell (dashed lines) described in Figure S1. Here, B and H are bridge and hollow sites on {111} step, respectively.

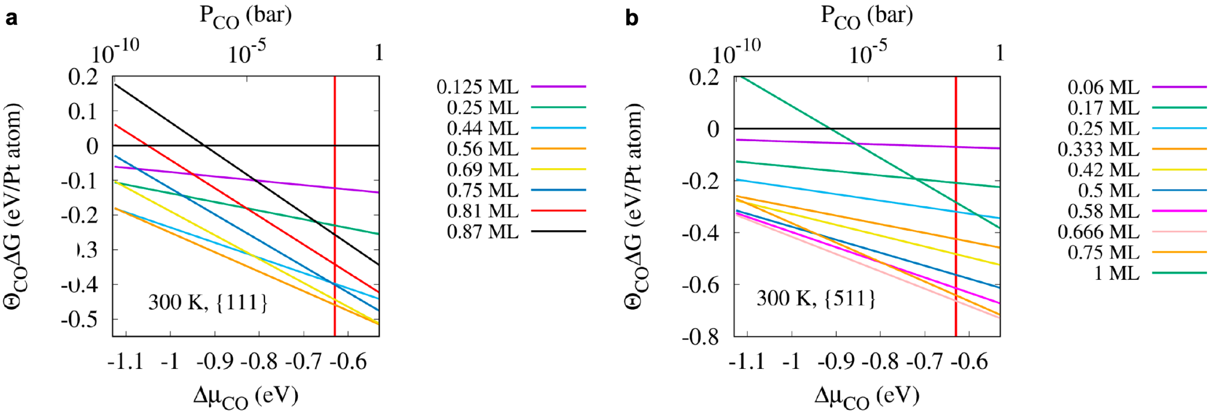

Supplementary Figure 23: Gibbs free energy of adsorption computed using revPBE as a function of the CO chemical potential at 300 K for the **a** {111} and the **b** {511} facets. The vertical lines correspond to 0.02 bar.

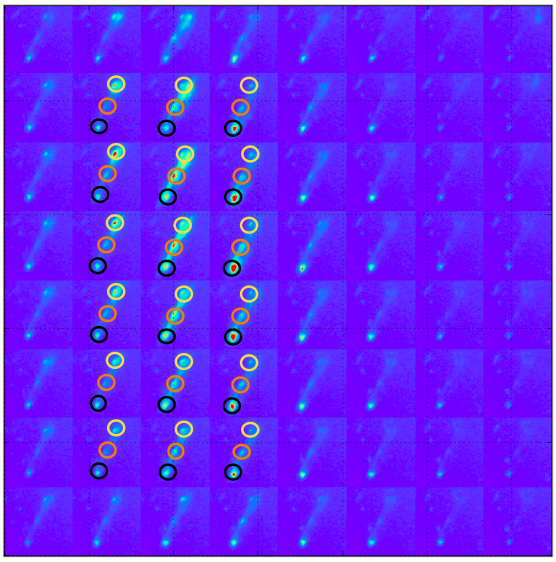

Supplementary Figure 24: Mosaic of two-dimensional Laue patterns measured around the **111** Laue spot of the crystal for different (*x*, *y*) positions of the sample (steps of 200 nm in *x* and *y*). Three peaks are observed and are indicated by circles of different colors. The crystal is thus constituted of at least three grains with an orientation close to [111].

References SI

[1] Hammer, B., Hansen, L. B. & Nørskov, J. K. Improved adsorption energetics within density-functional theory using revised perdew-burke-ernzerhof functionals. *Phys. Rev. B* **59**, 7413–7421 (1999).

[2] Patra, A., Bates, J. E., Sun, J. & Perdew, J. P. Properties of real metallic surfaces: Effects of density

functional semilocality and van der waals nonlocality. *Proceedings of the National Academy of Sciences* **114**, E9188–E9196 (2017).
[3] Tyson, W. & Miller, W. Surface free energies of solid metals: Estimation from liquid surface tension measurements. *Surface Science* **62**, 267–276 (1977).
[4] Tyson, W. Surface energies of solid metals. *Canadian Metallurgical Quarterly* **14**, 307–314 (1975).
[5] Gunasooriya, G. T. K. K. & Saeys, M. Co adsorption on Pt(111): From isolated molecules to ordered high-coverage structures. *ACS Catalysis* **8**, 10225–10233 (2018).
[6] Avanesian, T. et al. Quantitative and atomic-scale view of co-induced Pt nanoparticle surface reconstruction at saturation coverage *via* DFT calculations coupled with *in situ* TEM and IR. *Journal of the American Chemical Society* **139**, 4551–4558 (2017).
